# Age, sex, primary tumor type and site are associated with mortality after pathological fractures: an observational study of 1453 patients from the Swedish Fracture Register

**DOI:** 10.1186/s13018-023-03620-z

**Published:** 2023-03-01

**Authors:** Johan Wänman, Sonja Kjartansdóttir, Olof Wolf, Jonas Sundkvist, David Wennergren, Sebastian Mukka

**Affiliations:** 1 Department of Surgical and Perioperative Sciences (Orthopedics), Umeå University, Umeå, Sweden; 2 Institute of Clinical Sciences, Sahlgrenska Academy, University of Gothenburg, Gothenburg, Sweden; 3 Section of Orthopaedics, Department of Surgical Sciences, Uppsala University, Uppsala, Sweden

## Abstract

**Background:**

Pathological fractures are challenging in orthopedic surgery and oncology, with implications for the patient’s quality of life, mobility and mortality. The efficacy of oncological treatment on life expectancy for cancer patients has improved, but the metastatic pattern for bone metastases and survival is diverse for different tumor types. This study aimed to evaluate survival in relation to age, sex, primary tumor and site of the pathological fractures.

**Methods:**

All pathological fractures due to cancer between 1 September 2014 and 31 December 2021 were included in this observational study from the Swedish Fracture Register (SFR). Data on age, sex, tumor type, fracture site and mortality were collected.

**Results:**

A total of 1453 patients with pathological fractures were included (48% women, median age 73, range 18–100 years). Unknown primary tumors were the most common primary site (n = 308). The lower extremities were the most common site of pathological fractures. Lung cancer had the shortest median survival of 78 days (range 54–102), and multiple myeloma had the longest median survival of 432 days (range 232–629). The site at the lower extremity had the shortest (187 days, range 162–212), and the spine had the longest survival (386 days, range 211–561). Age, sex, primary type and site of the pathological fractures were all associated with mortality.

**Interpretation:**

Age, sex, primary tumor type and site of pathological fractures were associated with survival. Survival time is short and correlated with primary tumor type, with lung cancer as the strongest negative predictor of survival.

**Supplementary Information:**

The online version contains supplementary material available at 10.1186/s13018-023-03620-z.

## Introduction

Because the efficacy of treatment options available to cancer patients has improved, so has the patient’s average life expectancy following a cancer diagnosis [1]. Metastatic bone disease results in weakened and pathologic bone prone to a painful fracture, with considerable implications for patient quality of life (QoL), functionality and mortality [2, 3]. As such, metastatic long bone fractures cause a significant health care burden and are associated with poor functional outcomes and reduced life expectancy [3]. The humerus, femur and tibia are the most common targets of long bone metastasis, particularly from primary tumors originating from the breast, thyroid, kidney, lung and prostate [4]. Spinal metastases affect up to 70% of patients with cancer with a severe risk of para- or tetraplegia development due to metastatic spinal cord compression [5]. Orthopedic treatment of metastatic bone disease aims to alleviate pain and increase mobility and functional independence. The reconstruction must be durable in terms of life expectancy, avoiding lengthy stays in the hospital or long periods of rehabilitation, even when fracture healing may be doubtful [6–8]. Guiding clinical decision-making algorithms (e.g., PATHFx) provides valuable aid in predicting patient outcome [9]. However, extensive register-based studies describing the treatment and outcome after pathological fractures in all body parts are scarce [10]. The Swedish Fracture Register (SFR) offers unique opportunities to conduct large register-based studies on pathological fractures in all body parts, regardless of treatment.

This study aimed to describe the sex and age distribution, primary tumor types, site of pathological fractures and primary treatment. We also analyzed predictors of mortality in patients with pathological fractures using the SFR.

## Materials and methods

### Study design and setting

This observational register study was designed based on data derived from the SFR. Established in 2011, the SFR is a national quality register for managing and treating fractures [10]. Several studies have found the registration in the SFR to have high accuracy and validity [11, 12].

The proportion of departments affiliated with the SFR has increased gradually: in January 2014, 40% of affiliated departments were active. As of 1 January 2021, all orthopedic departments (n = 54) in Sweden are engaged in the SFR, resulting in 100% coverage. More than 650,000 fractures had been registered by the end of 2021.

The injury mechanism includes information on stress, spontaneous and pathological fractures.

The registration of pathological fractures (International Classification of Diseases, 10th Revision, ICD-10 M84.4.A-G) in the SFR includes specification of the primary tumor as breast, prostate, kidney, lung, myeloma, other or unknown. Treatment is registered with the chosen type of therapy (nonoperative or operative). Operative treatments consist of fracture fixation, including types of osteosynthesis (screws, pins, plates, sliding hip device, long and short intramedullary nails), arthroplasty (hemi or total, cemented or cementless fixation) or other (i.e., excision arthroplasty).

### Patient selection

All pathological fractures due to cancer (ICD code M 84.4) in adults registered in the SFR between 1 September 2014 and 31 December 2021 were included. A total of 1,577 pathological fractures were extracted from the SFR (ICD-10 M84.4.A-G). Of these 1,577 pathological fractures, 124 (7.9%) were excluded from further analysis because of multiple pathological fractures in the same patient. Only the first (index) pathological fracture was included.

### Outcome variables

Epidemiological data on age, sex, primary tumor type, site of the pathological fracture, treatment and mortality were analyzed.

### Statistics

Variables are presented as the proportion of all pathological fractures (%). Nominal variables are described as proportions of all fractures and scale variables as medians (range). Categorical variables were analyzed with the Chi-square test. The log-rank test was used for survival analysis. Cox proportional hazards with adjusted analyses were performed to evaluate overall mortality-related factors. A multivariate model adjusted for age, sex, site of pathological fracture (upper or lower extremity, spine or pelvic regions) and specification of the primary tumor (breast, prostate, kidney, lung, myeloma, other or unknown) were included in the analysis as covariates. The assumption of proportional hazards was investigated by introducing an interaction term of the covariates of interest with time and the finding that the interaction term was not statistically significant for all covariates. The associations are presented as hazard ratios (HRs) with 95% confidence intervals (CIs). A p-value < 0.05 was considered statistically significant. SPSS version 28 (IBM Corp, Armonk, NY, USA) was used for statistical analysis.

## Results

### Study patients and descriptive data

After exclusions, 1453 patients (48% women) were included*.* Both women (n = 703) and men (n = 750) had a median age of 73 (range 20–100 and 18–100, respectively) years. The sites of the pathological fractures were categorized as upper extremity (n = 484), pelvic (n = 74), spine (n = 132) and lower extremity (n = 763) (Table 1). The anatomical sites within each category are listed in (Additional file 1: Table S1).

**Table 1:** Demography and distribution of the pathological fractures

	Unknown	Breast	Prostate	Kidney	Lung	Other	Myeloma	Total
*Localization*								
Upper Extremity	107	65	62	33	44	77	96	484
Lower Extremity	152	137	173	29	77	122	73	763
Pelvic	13	6	23	4	6	19	3	74
Spine	36	8	28	6	10	24	20	132
Total	308	216	286	72	137	242	192	1453
*Sex (N)*								
Male	145	4	286	45	53	112	105	750
Female	163	212	0	27	84	130	87	703
Treatment*								
Operative	205	157	200	44	93	145	112	956
Non-operative	84	51	76	19	37	13	6	425
*Age* (Min–Max)	74 (18–100)	71 (35–100)	77 (53–98)	71 (24–95)	72 (49–97)	72 (37–96)	70 (18–95)	73 (18–100)

*72 patients missing data on treatment

#### Type and site of pathological fractures

Unknown primary tumors were the most common primary tumor (n = 308), followed by prostate (n = 286), other (n = 242), breast (N = 216), myeloma (n = 192), lung (n = 137) and kidney (n = 72) (Table 1). The lower extremities were the most common location for pathological fractures, followed by the upper extremities, spine and pelvic. The distribution was significantly different between the primary tumor types, with breast cancer having the highest proportional frequency of pathological fractures in the lower extremity (63%). Multiple myeloma had the highest proportional frequency among the pathological fractures in the upper extremity (50%), prostate cancer had the highest proportional frequency for pathological fractures in the pelvis (8%), and unknown primary tumors had the highest proportional frequency in the spine (12%) (Table 1).

#### Treatment

Operative treatment was performed in 956 (66%) patients and nonoperative treatment in 425 (29%). Missing information about treatment was noted in 72 (5%) patients. Operative treatment was performed in 93% of the fractures in the lower extremity, 6% in the pelvis, 36% in the spine and 43% in the upper extremity. 25 patients (6%), initially chosen for non-operative treatment (6 lower extremity, 1 spine, 1 pelvic and 17 upper extremity fractures), underwent surgery at a later stage.

#### Mortality

**Fig. 1:**
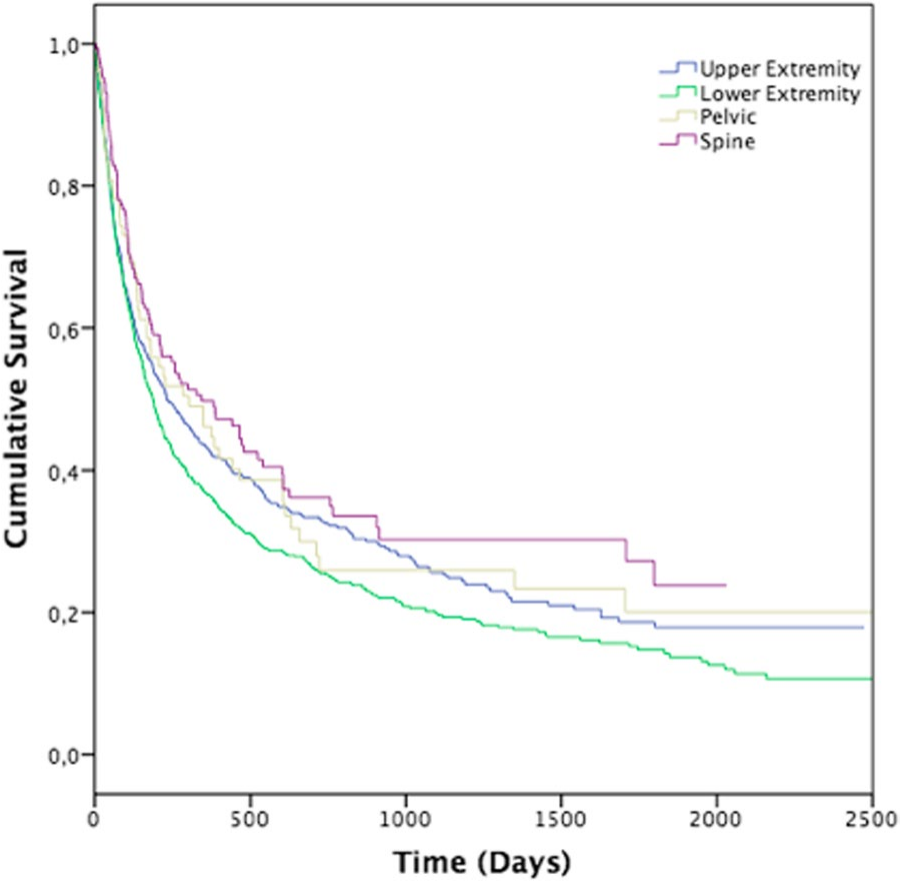
Survival function estimated by Kaplan–Meier method with mortality as an endpoint

Median survival was 213 days (95% CI, 185–241). Women had a median survival of 238 (range 188–288) days and men 199 (range 168–229). Lung cancer had the shortest median survival of 78 days (range 54–102), and multiple myeloma had the longest median survival of 432 days (range 232–629). The locations of the pathological fractures were of significant impact on survival, with the longest survival for spinal fractures (386 days, range 211–561) and the shortest survival for fractures of lower extremities (187 days, range 162–212) (Fig. 1). Mortality was 12% at 30 days, with the highest mortality rate for lung cancer (28%) and the lowest for multiple myeloma (6%). One-year mortality was 60%, with the highest rate for lung cancer (83%) and the lowest for multiple myeloma (45%), 209 patients had a follow-up less than one-year (Table 2 and 3).

**Table 2:** 30-day mortality

	Alive	Dead	Total	%
Unknown	277	31	308	10
Breast	194	22	216	10
Prostate	250	36	286	13
Kidney	63	9	72	13
Lung	98	39	137	28
Myeloma	180	12	192	6
Other	216	26	242	11
Total	1278	175	1453	12

**Table 3:** 1-year mortality. 1244 patients with a complete 1-year follow-up

	Alive	Dead	Total	%
Unknown	135	124	259	48
Breast	81	102	183	56
Prostate	80	166	246	67
Kidney	24	38	62	61
Lung	21	100	121	83
Myeloma	94	78	172	45
Other	75	126	201	63
Total	510	734	1244	59

In the multiple cox regression model, age, sex, primary tumor and site of the pathological fractures were significant for survival (Table 4).

**Table 4:** Cox proportional hazard model using myeloma, spine and being female as reference categories

	HR	95% CI	*P*-value
*Primary tumor*			
Unknown	1.05	0.83–1.32	0.695
Breast	1.53	1.19–1.97	0.001
Prostate	1.48	1.16–1.88	0.002
Kidney	1.46	1.06–2.00	0.021
Lung	3.19	2.47–4.12	< 0.001
Other	1.43	1.12–1.82	0.004
Myeloma	1	Ref	–
*Location of pathological fracture*			
Upper extremity	1.36	1.06–1.73	0.015
Lower extremity	1.38	1.09–1.75	0.007
Pelvic	0.98	0.69–1.39	0.907
Spine	1	Ref	–
Age (years)	1.03	1.02–1.03	< 0.001
*Sex*			
Male	1.18	1.01–1.38	0.04
Female	1	Ref	–

## Discussion

The main findings of this study are that age, sex, primary tumor and location of the pathological fractures were associated with survival. Unknown primary tumors and prostate cancer were the two most common primary tumors. The lower extremity was the most common site of pathological fractures. The pattern of the pathological fractures differed between the primary tumors and the median survival time was short.

### Primary site

Tumors behave differently in their affinity to seed metastasis to bones [13]. Primary tumors from breast, prostate, renal, lung and myeloma account for most bone metastases [4, 13]. However, an unknown primary tumor was the most common cause of pathological fracture in our study. It is essential to distinguish between an unknown primary tumor, defined as a histological malignancy for which no identifiable primary site can be found and a pathological fracture as a first manifestation before diagnostic workup is completed. Metastases with no identifiable primary tumor after diagnostic workup are generally classified as cancer of unknown primary. The cancer of unknown primary shares a characteristic aggressive course with rapid progression to metastasis, poor patient response to therapy and poor survival [14, 15]. In the SFR, however, there is no option to distinguish between these two, as registration in the SFR is done at the time of diagnosis and treatment of the pathological fracture before diagnostic workup of the primary tumor site has been fulfilled.

### The metastatic pattern

Two main hypotheses for anatomical distributions of metastasis have been proposed: the hemodynamic hypothesis and the seed and soil hypothesis. The hemodynamic hypothesis infers that metastasis is determined by anatomical vascular and lymphatic drainage [16]. The seed and soil hypothesis suggests that specific favorable interactions at the metastatic site are involved in the selection for metastatic growth [17]. The femur was the most common long bone affected by pathological fractures, followed by the humerus and tibia in concordance with previous reports [4]. Spinal metastases have previously been reported as the most frequent site of skeletal metastasis, accounting for approximately 70% of all bone metastases [18]. The differences observed could be attributed to an underreporting of pathological fractures in the spine. It is also possible that some spinal metastasis, even though treated surgically due to spinal cord compression, is not regarded as pathological fractures and therefore, not registered in the SFR. The specific mechanisms responsible for each tumor's preferable bone site remain to be investigated.

#### Treatment

Treatment of pathological fractures is generally palliative, aiming to reduce pain and improve patient functionality and QoL [6]. Most (93%) of the pathological fractures in the lower extremity were treated with surgery, compared to 43% in the upper extremity. Surgical stabilization of lower extremity fractures enables the patient to stay mobilized. Pathological upper extremity fractures can be stabilized by a cast or brace, giving the patient some pain relief.

#### Mortality

The generally short survival for patients with pathological fractures presented in our study reflects the poor prognosis of pathological fractures. The poor prognosis calls for careful consideration of the potential benefits of surgical treatment contra the risk of complications and lengthy hospital stays. Our survival data align with some studies [3, 8, 14] and are shorter than previous cohorts for surgically treated pathological fractures [19]. The shorter survival could, in part, be attributed to selection bias, i.e., patients selected for surgery may have a longer life expectancy. Our study included all pathological fractures, operatively and nonoperatively treated. Survival was associated with age, sex, primary tumor and site of the fracture. The primary tumor has previously been reported as a major predictor of survival [3]. Patients with myeloma, lymphoma and breast and kidney cancer have reported a higher 1-year survival rate, whereas patients with lung, unknown primaries and prostate cancer have a lower survival rate at 1-year [4, 14, 20]. Consistent with a recent study, lung cancer has been reported as an independent negative predictor of survival, whereas myeloma was a favorable prognostic factor for survival [4, 20]. Differences in survival related to heterogeneous types and responses to oncological treatment have been reported, even within the same group of tumors [21, 22]. The anatomical location of the pathological fractures significantly impacted survival, and patients with pathological fractures in the lower extremity had the worst prognosis. The independent prognostic value for segment location of pathological fractures has, to our knowledge not been demonstrated previously. It is reasonable to assume that a pathological fracture to the lower extremity renders more prolonged immobilization than a pathological fracture in the upper extremity, although a larger share of them are surgically stabilized. This reasoning is supported by Bergh et al., who found that all types of femur fracture resulted in high mortality in a large register-based study on mortality [23]. Despite the worse prognosis for lower extremity locations, surgical treatment is probably reasonable in most cases to regain weight bearing and ambulation. In agreement with other studies, age was a predictor of survival [24].

### Strength and limitations

The main strength of this study is the large sample size of pathological fractures, including data on segment location and type of primary cancer. The independent prognostic value for segment location of pathological fractures has, to our knowledge, not been demonstrated. Using data from a national health care quality register with registration and classification of fractures entered by the treating physician enables the description of demographics, location, treatment choice and analysis of mortality. However, given the stepwise introduction and the present completeness of the SFR, we cannot identify the overall incidence, which could affect the external validity of the results [10]. The main limitations are linked to the retrospective study design and inherited limitations of register-based studies, including miscoding, transferring errors, under-reporting and missing data. Another limitation is that all metastasis to bone does not cause pathological fractures or are treated before a pathological fracture occurs. Impending fractures and known metastasis of primary tumors affect patients differently. These patients are not registered in the SFR. Thus, the current study covers pathological fractures but not all metastasis to bone. As mentioned earlier in the discussion, some unknown primary tumors might later have been correctly diagnosed. This information would not have been available to the treating physician entering data into the SFR at the time of the fracture.

## Conclusion

Age, sex, primary tumor site and location of pathological fractures were associated with survival. Survival time is short and correlated with primary tumors, with lung cancer as the strongest negative predictor of survival.

## Supplementary Information

**Additional file 1**: **Table S1.** Anatomical distribution within each segment of the pathological fractures.

## Data Availability

The dataset analyzed in this study is not publicly available as the study was approved on the grounds of ensuring the confidentiality of patient-identifiable information. We are positive to sharing data but are legally restricted from sharing the data publicly according to the law on Public Access and Secrecy, chapter 21, paragraph 7 and chapter 25, paragraph 1 (https://www.riksdagen.se/sv/dokument-lagar/dokument/svensk-forfattningssamling/offentlighets--och-sekretesslag-2009400_sfs-2009-400). Any person interested in the data set may contact Umeå University hospital and the corresponding author to find ways to share data according to Swedish laws and regulations. It is also possible for individuals interested in this data to apply directly from the Center of Registers, Västra Götaland (URL: http://registercentrum.se/). This process involves approval from the Swedish Ethical Review Authority.

## References

[1] Jemal A, Ward EM, Johnson CJ, Cronin KA, Ma J, Ryerson B (2017). Annual report to the nation on the status of cancer, 1975–2014, featuring survival. J Natl Cancer Inst..

[2] Narazaki DK, de Alverga Neto CC, Baptista AM, Caiero MT, de Camargo OP (2006). Prognostic factors in pathologic fractures secondary to metastatic tumors. Clinics.

[3] Kirkinis MN, Lyne CJ, Wilson MD, Choong PF (2016). Metastatic bone disease: a review of survival, prognostic factors and outcomes following surgical treatment of the appendicular skeleton. Eur J Surg Oncol.

[4] Ratasvuori M, Wedin R, Keller J, Nottrott M, Zaikova O, Bergh P (2013). Insight opinion to surgically treated metastatic bone disease: Scandinavian sarcoma group skeletal metastasis registry report of 1195 operated skeletal metastasis. Surg Oncol.

[5] Jaipanya J, Chanplakorn P (2022). Spinal metastases: narrative reviews of the current evidence and treatment modalities. J Int med Res.

[6] Anract P, Biau D, Boudou-Rouquette P (2017). Metastatic fractures of long limb bones. Orthop Traumatol Surg Res.

[7] Errani C, Mavrogenis AF, Cevolani L, Spinelli S, Piccioli A, Maccauro G (2017). Treatment for long bone metastases based on a systematic literature review. Eur J Orthop Surg Traumatol..

[8] Forsberg JA, Wedin R, Boland PJ, Healey JH (2017). Can we estimate short- and intermediate-term survival in patients undergoing surgery for metastatic bone disease?. Clin Orthop Relat Res.

[9] Anderson AB, Wedin R, Fabbri N, Boland P, Healey J, Forsberg JA (2020). External validation of PATHFx version 3.0 in patients treated surgically and nonsurgically for symptomatic skeletal metastases. Clin Orthop Relat Res..

[10] Möller M, Wolf O, Bergdahl C, Mukka S, Rydberg EM, Hailer NP (2022). The Swedish Fracture Register—ten years of experience and 600,000 fractures collected in a National Quality Register. BMC Musculoskelet Disord..

[11] Knutsson SB, Wennergren D, Bojan A, Ekelund J, Möller M (2019). Femoral fracture classification in the Swedish Fracture Register—a validity study. BMC Musculoskelet Disord..

[12] Wennergren D, Stjernström S, Möller M, Sundfeldt M, Ekholm C (2020). Validity of humerus fracture classification tin the Swedish fracture register. BMC Musculoskelet Disord..

[13] Macedo F, Laderia K, Pinho F, Saraiva N, Bonito N, Pinto L (2017). Bone metastases: an overview. Oncol Rev.

[14] Piccioli A, Maccauro G, Spinelli MS, Biagini R, Rossi B (2015). Bone metastases of unknown origin: epidemiology and principles of management. J Orthop Traumatol..

[15] Takagi T, Katagiri H, Kim Y, Suehara Y, Kubota D, Akaike K (2015). Skeletal metastasis of unknown primary origin at the initial visit: A retrospective analysis of 286 cases. Plos One.

[16] Ewing J (1928). Neoplastic disease. A treatise on tumors. Am J Med Sci.

[17] Paget S (1889). The distribution of secondary growths in cancer of the breast. Lancet.

[18] Delank KS, Wendtner C, Eich HT, Eysel P (2011). The treatment of spinal metastases. Dtsch Arztebl Int.

[19] Nathan SS, Healey JH, Mellano D, Hoang B, Lewis I, Morris CD (2005). Survival in patients operated on for pathologic fracture: implications for end-of-life orthopedic care. J Clin Oncol.

[20] Hansen BH, Keller J, Laitinen M, Berg P, Skjeldal S, Trovik C (2004). The Scandinavian Sarcoma Group skeletal metastasis register: survival after surgery for bone metastases in the pelvis and extremities. Acta Orthop Scand Suppl.

[21] Schneiderbauer MM, von Knoch M, Schleck CD, Harmsen WS, Sim FH, Scully SP (2004). Patient survival after hip arthroplasty for metastatic disease of the hip. J Bone Joint Surg Am..

[22] Lin PP, Mirza AN, Lewis VO, Cannon CP, Shi-Ming T, Nizar MT (2007). Patient survival after surgery for osseous metastases from renal cell carcinoma. J Bone Joint Surg Am.

[23] Bergh C, Möller M, Ekelund J, Brisby H (2022). Mortality after sustaining skeletal fractures in relation to age. J Clin Med.

[24] Weiss RJ, Tullberg E, Forsberg JA, Bauer HC, Wedin R (2014). Skeletal metastases in 301 breast cancer patients: patient survival and complications after surgery. Breast.

