# Supplementary figures and images for: Age, sex, primary tumor type and site are associated with mortality after pathological fractures: an observational study of 1453 patients from the Swedish Fracture Register
Source: J Orthop Surg Res. 2023 Mar 1;18:150. doi: 10.1186/s13018-023-03620-z (PMC9976455; doi:10.1186/s13018-023-03620-z)

**Supplementary Table 1. Localization of pathological fracture.**


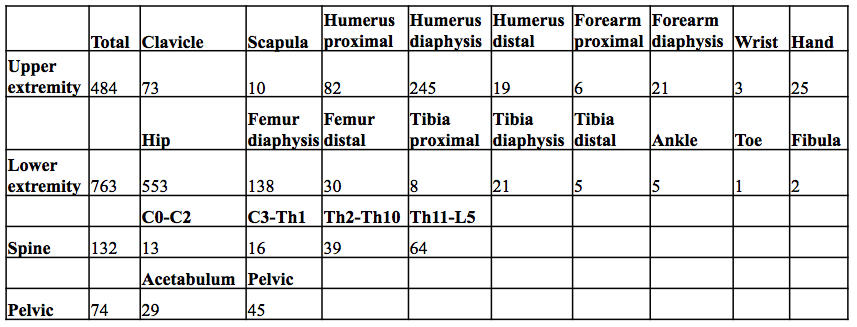

Supplement: Supplementary file 1 — Additional file 1: Table S1. Anatomical distribution within each segment of the pathological fractures. [file 13018_2023_3620_MOESM1_ESM.docx]
